# Excessive Daytime Sleepiness Is Associated With Non-motor Symptoms of Multiple System Atrophy: A Cross-Sectional Study in China

**DOI:** 10.3389/fneur.2021.798771

**Published:** 2022-01-11

**Authors:** Hui Wang, Xiangdong Tang, Junying Zhou, Yanming Xu

**Affiliations:** ^1^Department of Neurology, The Second People's Hospital of Chengdu, Chengdu, China; ^2^Sleep Medicine Center, West China Hospital, Sichuan University, Chengdu, China; ^3^Department of Neurology, West China Hospital, Sichuan University, Chengdu, China

**Keywords:** excessive daytime sleepiness, motor symptoms, non-motor symptoms, sleep parameters, multiple system atrophy

## Abstract

**Objectives:** Excessive daytime sleepiness (EDS) in multiple system atrophy (MSA) has received scant attention in the literature, thus the present cross-sectional study aimed to investigate the prevalence of EDS and its potential risk factors among Chinese patients with MSA.

**Methods:** A total of 66 patients with MSA (60.6% males) were consecutively recruited. Eighteen patients (27.3%, 13 men) with Epworth Sleepiness Scale score >10 were defined as having EDS. Demographic, motor [Unified Multiple-System Atrophy (UMSARS)] and non-motor symptoms [Non-Motor Symptoms Scale (NMSS)], and sleep parameters [polysomnography (PSG)] were compared between patients with MSA with and without EDS. A logistic regression analysis was used to calculate the risk factors of EDS in patients with MSA.

**Results:** There were no significant differences in age, sex, MSA onset age, disease duration, MSA sub-type, and motor symptom severity between MSA patients with and without EDS. However, compared with the MSA patients without EDS, their counterparts with EDS had higher scores of NMSS (65.3 ± 23.1 vs. 43.4 ± 25.3, *P* = .0002), Hamilton Anxiety (HAMA) [15.3 (10.3–20.0) vs. 9.5 (3.0–15.0), *P* = 0.006], Hamilton Depression (HAMD) [13.7 (12.5–17.8) vs. 9.0 (4.0–13.0), *P* = 0.015], and Fatigue Severity Scale (FSS) [29.8 (17.3–47.8) vs. 18.7 (10.3–21.8), *P* = 0.040]. Conversely, the patients with EDS had lower score of Mini-Mental State Examination (MMSE) [23.3 (20.3–27.0) vs. 25.7 (22.0–29.0), *P* = 0.023]. Similarly, there was a significantly lower percentage of N3 sleep (%) [0.3 (0–0) vs. 2.0 (0–0), *P* = 0.007] and a higher apnea-hypopnea index (AHI/h) [30.5 (14.5–47.8) vs. 19.3 (5.0–28.7), *P* = 0.034] in patients with EDS. After adjusting for age, sex, disease duration, MSA sub-type, and UMSARS score, the odds ratio (OR) (95% CI) of EDS was higher while increasing scores in FSS [1.06 (1.02–1.11)], HAMA [1.16 (1.04–1.28)], HAMD [1.13 (1.02–1.25)], NMSS [1.04 (1.01–1.07)], and AHI [1.03 (1.00–1.10)]. The OR of EDS was lower while the MMSE score was increasing [0.85 (0.72–1.00)].

**Conclusions:** The presence and severity of EDS may be significantly associated with the non-motor dysfunction, including fatigue, anxiety, depression, cognitive dysfunction, and sleep-related breathing disorder, but not with the motor dysfunction in MSA.

## Introduction

Multiple system atrophy (MSA) is defined as an episodic and rapidly progressing neurodegenerative disease characterized by Parkinsonism, autonomic dysfunction, and/or ataxia. According to its clinical manifestations, MSA is classified into MSA-P in the event that the patient presents with Parkinsonism symptoms and MSA-C if the patient develops cerebellar dysfunction (1). Currently, non-motor symptoms are well-known to have a significant impact on the quality of life as well as the motor symptoms among patients with MSA (2). Excessive daytime sleepiness (EDS) is a common non-motor symptom in patients with MSA. The previously reported prevalence of EDS in patients with MSA was 24–28% (2, 3), which was significantly higher than the prevalence of 2% in the healthy control group (2). EDS was considered as the result of inadequate sleep or sleep-related breathing disorder (SRBD) in MSA (2–4), but the most relevant cause might be the neural systems pathophysiological impairment, including cholinergic neurons in the mesopontine tegmentum, hypocretin/orexin neurons of the lateral hypothalamus, serotonergic neurons of the rostral raphe, and dopaminergic neurons in the ventral periaquapular gray matter (5–7).

A series of studies has found that EDS might be a preclinical marker of Parkinson's disease (PD) (8–10). In particular, a longitudinal study revealed that EDS increases with disease progression in PD (8, 9), which was considered as the degeneration of the lower brain stem involved in sleep-wake regulation. Although MSA is also classified into synucleinopathies as well as PD, there were only two studies concerned with the association between EDS and MSA until now. In previous studies, EDS was found to be weakly correlated with the severity of motor symptoms in patients with MSA (2, 4). However, there is no literature about differences in clinical features, such as the motor and non-motor symptoms between MSA patients with and without EDS. Thus, the present study investigates the prevalence of EDS among Chinese patients with MSA and compares the motor and non-motor symptoms between MSA patients with and without EDS. Furthermore, we explored the potential influencing factors on EDS among patients with MSA.

## Methods

### Participants

The present cross-sectional study enrolled 66 consecutive patients with “probable MSA” from the Department of Neurology of West China Hospital between 2017 and 2020. The diagnosis of MSA was based on the standard criteria of the Second consensus statement (2008) by neurologists (11). Patients were included if they (1) met the diagnostic criteria for probable MSA, (2) completed the assessments of subjective questionnaire scales and polysomnography (PSG), (3) and showed no signs of intracranial lesion on brain magnetic resonance imaging. Patients were excluded if they (1) had signs or symptoms of other neurologic diseases; (2) used medications or substances that might affect their sleep cycle, such as hypnotic drugs, antidepressants, selective serotonin reuptake inhibitors, and antipsychotic agents; or (3) were critically ill or presented unstable vital signs. The study was approved by the West China Hospital Clinic Research Ethics Committee, and all patients and their guardians provided written informed consent.

### Questionnaires

All patients were evaluated using several questionnaires validated in Chinese, and all the questionnaires were rated by clinicians. Epworth Sleepiness Scale (ESS) was used to assess subjective daytime sleepiness. EDS was defined as an ESS score > 10 (12). Sleep quality was assessed by the Pittsburgh Sleep Quality Index (PSQI). The Rapid Eye Movement (REM) Sleep Behavior Disorder Questionnaire (RBDSQ) was used to diagnose REM-sleep behavior disorder (RBD) (13). The Non-Motor Symptoms Scale (NMSS) (nine domains) was used to evaluate the severity of the non-motor symptoms. The symptoms of depression and anxiety were assessed using the Hamilton Depression Rating Scale (17 items) and Hamilton Anxiety Rating Scale (HAMA), respectively. Patients were evaluated for fatigue using the Fatigue Severity Scale (FSS). The Mini-Mental State Examination (MMSE) was used to evaluate cognitive function. The severity of motor symptoms was assessed using the Unified Multiple System Atrophy Rating Scale (UMSARS).

### PSG

All recruited patients underwent an overnight video-PSG assessment in the Sleep Medicine Center of West China Hospital. The continuous recordings included electroencephalography (F4–M1, C4–M1, O2–M1, F3–M2, C3–M2, O1–M2), electrooculography (ROC–M1, LOC–M2), submental electromyography, right and left anterior tibialis surface electromyography, and electrocardiography. Other measurements included oxygen saturation, nasal-oral flow, thoracic and abdominal respiratory efforts, and body position. The PSG results were scored by sleep technicians and interpreted by sleep specialists. Sleep stages and associated events were manually scored in 30-s epochs, according to the guidelines for scoring sleep and related events published by the American Academy of Sleep Medicine (14). The diagnosis of sleep-related breathing disorder (SRBD) was based on PSG measurement with the Apnea-Hypopnea Index (AHI) >5/h (15).

### Statistical Analysis

Data were analyzed using SPSS 19.0 (IBM, Chicago, IL, USA). Continuous data showing normal distribution were expressed as the mean ± SD, while continuous skewed data were reported as the median (interquartile range). Group differences were calculated using Student's *t*-test or the Mann-Whitney *U*-test for continuous variables. Differences in categorical data were calculated by the Chi-square test. Logistic regression analysis was used to estimate the odds ratio (OR) and 95% CI after adjusting for potential confounding factors, including quantitative variables (age, disease duration, UMSARS score) and categorical variables (sex, MSA sub-type). Values of *p* < 0.05 were considered to indicate statistical significance.

## Results

This study included 66 patients with probable MSA (40 men), and 27.3% of patients (*n* = 18) were diagnosed with EDS based on ESS score >10 (12). The mean ESS score of the patients with MSA wasere 6.0 (2.8–11.0). Among the patients with MSA, there were 47 MSA-P (14 with EDS) and 19 MSA-C (4 with EDS) subtypes. There was no significant difference in the prevalence of EDS between patients with MSA-C and MSA-P (*P* = 0.643). In addition, the prevalence of SRBD in MSA patients was 78.8% (52/66) and no significant difference was found in the rates of SRBD between patients with MSA-C (89.5%) and MSA-P (74.5%).

### Demographic and Clinical Characteristics

The comparisons of demographic and clinical characteristics between patients with and without EDS are presented in Table 1. There were no significant differences in age, MSA onset age, disease duration, UMSARS, RBDSQ, and PSQI scores between the two groups. However, compared with the MSA patients without EDS, their counterparts with EDS had higher scores of FSS, NMSS, HAMA, and HAMD. Conversely, the patients with EDS had lower MMSE scores (*P* < 0.05).

**Table 1:** Demographic and clinical characteristics of multiple system atrophy (MSA) patients with and without excessive daytime sleepiness (EDS).

	**Total**	**MSA with EDS**	**MSA without EDS**	***P***
	**(*n* = 66)**	**(*n* = 18)**	**(*n* = 48)**	
Age, y	63.1 (54.8–68.3)	64.8 (59.8–72.5)	62.4 (54.0–66.8)	0.334
Male/female	40/26	13/5	27/21	0.237
Age at onset, y	60.2 ± 10.3	62.0 ± 9.6	59.4 ± 10.6	0.369
Disease duration, y	2.8 (1.0–3.0)	2.5 (1.0–3.1)	2.9 (1.1–3.0)	0.959
MSA sub-type (P/C)	47/19	14/4	33/14	0.643
ESS	6.0 (2.8–11.0)	14 (12.0–17.3)	3.0 (2.0–6.0)	**0.000**
PSQI	6.6 (3.0–9.5)	7.8 (3.8–10.0)	6.2 (3.0–8.0)	0.099
RBDSQ	4.7 (1.5.0–7.5.0)	5.7 (2.0–9.0)	4.3 (1.0–6.0)	0.110
FSS	21.7 (11.0–30.0)	29.8 (17.3–47.8)	18.7 (10.3–21.8)	**0.040**
NMSS	49.4 ± 26.4	65.3 ± 23.1	43.4 ± 25.3	**0.002**
MMSE	25.0 (22.0–29.0)	23.3 (20.3–27.0)	25.7 (22.8–29.0)	**0.023**
HAMA	11.1 (4.0–17.5)	15.3 (10.3–20.0)	9.5 (3.0–15.0)	**0.006**
HAMD	10.3 (4.0–15.0)	13.7 (12.5–17.8)	9.0 (4.0–13.0)	**0.015**
UMSARS I	13.23 ± 5.8	14.8 ± 5.7	12.7 ± 5.7	0.182
UMSARS II	16.5 (12.0–20.3)	18.61 (15.0–21.5)	15.8 (12.0–20.0)	0.083
UMSARS IV	1.7 (1.0–2.0)	1.9 (1.0–3.0)	1.7 (1.0–2.0)	0.257

*ESS, Epworth Sleepiness Scales; FSS, Fatigue Severity Scale; HAMD, Hamilton Depression Scale; HAMA, Hamilton Anxiety Scale; MSA, Multiple system atrophy; MMSE, Mini-mental State Examination; MSA sub-type (P/C), MSA sub-type (Parkinsonism/cerebellar ataxia); NMSS, Non-Motor Symptoms Scale; PSQI, Pittsburgh Sleep Quality Index; RBDSQ, Sleep Behavior Disorder Questionnaire; UMSARS, Unified Multiple-System Atrophy Rating Scale. Values are n (%), mean ± SD or median (interquartile range). The bold value of P value are P < 0.05*.

The comparisons of the subitems of the NMSS scale between MSA patients with and without EDS are shown in Table 2. Patients with EDS had higher scores for sleep/fatigue, mood disorder, perceptual problems, attention/memory dysfunction, gastrointestinal dysfunction, unexplained pain, and weight changes.

**Table 2:** Non-motor symptoms scale (NMSS) of MSA with and without EDS.

	**Total**	**MSA with EDS**	**MSA without EDS**	***P***
	**(*n* = 66)**	**(*n* = 18)**	**(*n* = 48)**	
Cardiovascular	1.9 (0–3.3)	3.0 (0–4.5)	1.5 (0–1)	0.335
Sleep/fatigue	7.0 (2.0–12.0)	11.8 (8.0–16.0)	5.2 (1.0–8.8)	**0.000**
Mood/apathy	8.1 (0.8–12.0)	12.3 (5.0–15.3)	6.5 (0–11.8)	**0.007**
Perceptual problems	1.5 (0–1.3)	2.5 (0–4.3)	1.1 (0–0.8)	**0.036**
Attention/memory	3.7 (0–7.0)	5.9 (2.8–8.0)	2.9 (0–5.0)	**0.005**
Gastrointestinal	4.5 (0–8.0)	6.4 (2.0–9.5)	3.7 (0–6.0)	**0.041**
Urinary	9.7 (4–15.3)	8.6 (0.8–12.3)	10.4 (4.0–16.8)	0.750
Sexual dysfunction	8.4 (0–15.0)	8.6 (2.8–18.0)	8.3 (0–14.3)	0.484
Miscellaneous	4.6 (0–7.3)	6.3 (3–9.3)	3.9 (0–6.0)	**0.036**
Pain	1.0 (0–0)	2.0 (0–4.0)	0.63 (0–0)	**0.028**
dysgeusia/ dysosmia	1.8 (0–2.0)	2.1 (0–4.5)	1.8 (0–1.5)	0.582
weight	0.23 (0–0)	0.7 (0–1.3)	0.04 (0–0)	**0.004**
desudation	1.48 (0–3.0)	1.5 (0–3.3)	1.5 (0–2.3)	0.454

*NMSS, Non-Motor Symptoms Scale. Values are median (interquartile range). The bold value of P value are P < 0.05*.

### Sleep Parameters

The comparisons of PSG parameters between MSA patients with and without EDS are shown in Table 3. No significant differences were found between MSA patients with and without EDS in terms of total sleep time (TST), sleep efficiency (SE), sleep latency (SL), the percentages of N1, N2, and REM sleep stage, minimum SaO_2_, and periodic leg movement index. However, there were significant differences in sleep-breathing-related variables between the two groups. Compared with the MSA without EDS group, the MSA with EDS group had significantly higher AHI, central apnea index (CAI), and hypopnea index (HI). Similarly, there was a slightly higher obstructive apnea index (OAI) in patients with EDS. In addition, the MSA with EDS group had a lower percentage of N3 sleep.

**Table 3:** Comparisons of sleep parameters between MSA patients with and without EDS.

	**Total**	**MSA with EDS**	**MSA without EDS**	***P***
	**(*n* = 66)**	**(*n* = 18)**	**(*n* = 48)**	
TST, min	337.0 ± 90.2	350.8 ± 80.4	331.8 ± 93.9	0.449
SE, %	66.0 ± 17.3	68.0 ± 16.0	65.2 ± 17.9	0.277
SL, min	22.9 (5–28)	16.2 (5.4–23.1)	25.4 (5.0–29.0)	0.708
N1, %	32.2 (30.6–42.35)	35.4 (20.9–48.7)	30.9 (17.5–40.2)	0.163
N2, %	48.1 ± 14.6	45.2 ± 14.0	49.2 ± 14.8	0.333
N3, %	1.5 (0–0.32)	0.3 (0–0)	2.0 (0–0)	**0.007**
REM, %	18.3 ± 3.8	19.1 ± 6.9	18.0 ± 8.1	0.614
WASO, min	151.1 ± 83.9	150.3 ± 85.3	151.4 ± 84.2	0.962
Arousal index/h	19.7 (13.6–23.6)	18.6 (10.6–22.4)	20.1 (11.5–24.8)	0.746
AHI/h	22.3 (6.1–32.7)	30.5 (14.5–47.8)	19.3 (5.0–28.7)	**0.034**
OAI/h	7.4 (0–9.0)	12.8 (0–34.8)	5.8 (0–5.5)	0.080
CAI/h	0.4 (0–0.4)	1.8 (0–0.3)	1.0 (0–1.2)	**0.016**
HI/h	11.7 (3.4–17.3)	17.3 (8.6–20.6)	9.8 (2.6–15.2)	**0.018**
Minimum SaO_2_, %	85.4 (83–90)	84.2 (79.8–90.0)	85.9 (84.3–90.0)	0.323
PLMI/h	25.2 (4.3–43.5)	21.9 (4.3–36.60)	26.3 (4.1–44.6)	0.619

*AHI, apnea–hypopnea index; CAI, Central apnea index; EDS, excessive daytime sleepiness; HI, hypopnea index; MSA, Multiple system atrophy; OAI, obstructive apnea index; REM, rapid eye movement; SaO_2_, oxygen saturation; SE, sleep efficiency; SL, sleep latency; TST, total sleep time. Values are n (%), mean ± SD or median (interquartile range). The bold value of P value are P < 0.05*.

### Risk Factors of EDS

**Figure 1:**
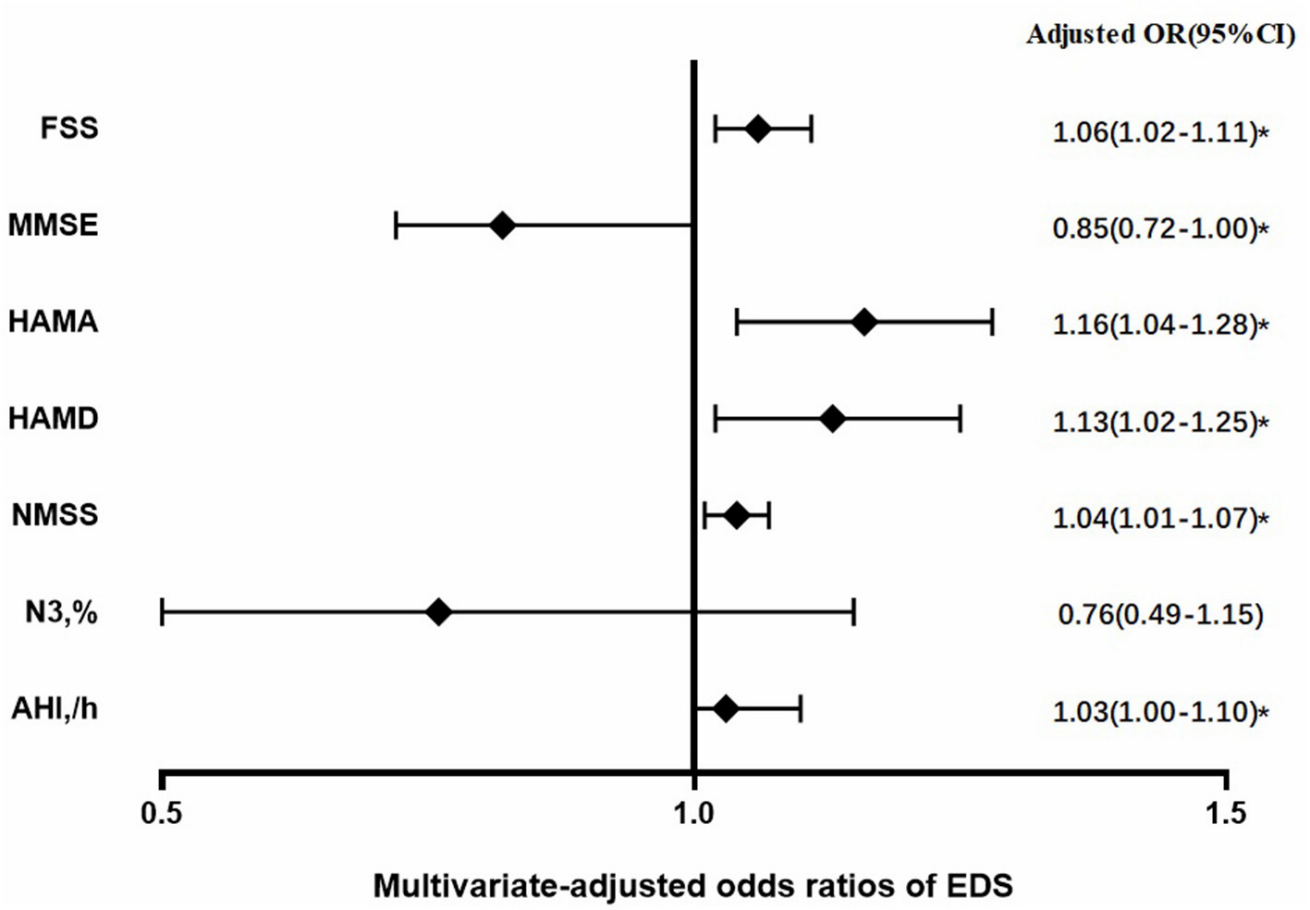
Risk factors of excessive daytime sleepiness (EDS) in patients with multiple system atrophy (MSA) after adjustment for age, sex, disease duration, MSA sub-type, and UMSARS score; OR, odds ratio; CI, confidence interval; *P < 0.05.

Figure 1 shows the risk factors of EDS in patients with MSA. After adjusting for age, sex, disease duration, MSA subtype, and UMSARS score, the risk of EDS in patients with MSA were significantly higher while increasing scores in FSS [1.06 (1.02–1.11)], HAMA [1.16 (1.04–1.28)], HAMD [1.13 (1.02–1.25)], NMSS [1.04 (1.01–1.07)], and AHI [1.03 (1.00–1.10)]. Conversely, the risk of EDS in patients with MSA was significantly lower while the MMSE score was increased [0.85 (0.72–1.00)]. Supplementary Table 1 presents the process of logistic regression analysis. Therefore, the risk of EDS in patients with MSA is associated with more severe symptoms of fatigue, anxiety, depression, cognitive dysfunction, and SRBD.

## Discussion

As far as we know, there is little literature investigating the differences of motor and non-motor symptoms between MSA patients with and without EDS. The present study found that the prevalence of EDS was 27.3% among patients with MSA, which is rather similar to those previously reported in Japan (24%) and in Europe (28%) (2, 4). Notably, a previous study showed that the incidence of EDS in MSA was similar to that of patients with PD (28 and 29%, respectively).Thus, these findings indicated that EDS is relatively common in patients with MSA as well as other synucleinopathies like PD.

REM-sleep behavior disorder (RBD) is characterized by the loss of muscular atonia and prominent motor behavior during REM sleep, which is a common symptom in patients with MSA (16). The prevalence of RBD is up to 69–100% in patients with MSA (17–19). Therefore, RBD is considered as a “red flag” which is associated with the faster progression and greater severity of synucleinopathies including PD and MSA (20). This study investigates the association between EDS and clinical symptoms including motor and non-motor symptoms and addressed the question of whether EDS—like RBD—aggravates the progression of MSA (20). Finally, we found there were no differences in age, disease duration, MSA sub-type, and UMSARS scores between the groups with and without EDS. Similarly, a previous study reported that there was no correlation between EDS and the severity of motor symptoms in patients with MSA (2). Even so, a larger study is necessary to determine the relationship between EDS and motor dysfunction in MSA.

In the current study, a significantly higher score of NMSS was found in MSA patients with EDS, especially in sleep/fatigue, mood/apathy, perceptual problems, attention/memory, gastrointestinal, pain, and weight dimensions. Moreover, MSA patients with EDS had higher scores of HAMA and HAMD than those without EDS. Previous studies showed that the risk of EDS was increased by the level of depression (21, 22). This indicated that the symptoms of depression and anxiety might increase the incidence and severity of EDS in patients with MSA, meanwhile, EDS also might deteriorate the symptoms of depression and anxiety in patients with MSA. Our findings suggested that more attention should be paid to MSA patients with EDS and timely intervention on mood disorders might alleviate patients' daytime sleepiness.

Our findings suggested that the severity of fatigue may increase the risk of EDS and lead to more severe EDS. A previous study found that EDS and fatigue are common in patients with MSA and there was a significant correlation between fatigue and EDS (23). Therefore, the interaction between fatigue and EDS indicated that EDS may deteriorate the symptom of fatigue, on the other hand, fatigue may increase the risk of EDS. EDS was reported to be common in the patients of dementia with Lewy bodies (DLB) and PD with dementia (24), other studies also found that EDS could influence cognitive function and increase the risk of dementia (25, 26). Similarly, in the present study, we found more severe cognitive impairment might increase the risk of EDS in patients with MSA. Future studies with larger sample size and follow-up observation are needed to elucidate the relationship between EDS and cognitive function in patients with MSA.

Our study found that MSA patients with EDS had a worse gastrointestinal function and weight loss. Gastrointestinal dysfunction is one of the common autonomic dysfunctions (AUD) in synucleinopathies including MSA, PD, DLB, and pure autonomic failure (27). Patients with MSA manifested severe autonomic dysfunction due to severe impairment in the central and peripheral autonomic networks (28). Our findings suggested that MSA patients with EDS could have more widespread impairment in the central and peripheral autonomic networks than those without EDS. In addition, the effect of gastrointestinal dysfunction can partially underlie the worsened weight loss in patients with MSA with EDS.

Sleep-related breathing disorder, including central sleep apnea, obstructive sleep apnea, and hypopnea, is common sleep disorder in patients with PD or MSA (29). Previous studies have shown that ~15–70% of patients with MSA develop sleep-related breathing disorders (6, 7, 30). Our results showed that MSA patients with EDS had a higher prevalence of central sleep apnea and hypopnea compared to those without EDS. Furthermore, the results of logistic regression analysis indicated that more severe sleep-breathing disorders might result in a higher risk of EDS in MSA. This finding is consistent with the consensus that EDS may be stronger associated with SRBD (31). In addition, we found the percentage of N3 sleep was significantly lower in MSA patients with EDS than that without EDS. The reduction of the N3 stage might be due to the more severe SRBD in MSA patients with EDS.

The strengths of the present study included the considerable sample size and the comprehensive clinical and PSG variables. However, several limitations should be noted in this study. First, the diagnosis of MSA was based on clinical assessment and no autopsy confirmed the diagnosis, but we strictly followed the standard diagnostic criteria to ensure accuracy and consistency. Second, all patients that we included were required to have met the diagnostic criteria of probable MSA, which may have contributed to a selection bias toward more severe cases. Third, patients who took medicines such as hypnotic drugs antidepressants were excluded, which may potentially mean that patients with severe non-motor symptoms were excluded. Fourth, the EDS diagnosis was based on a questionnaire but no objective assessments of sleepiness, such as the multiple sleep latency test (MSLT). Finally, this is a cross-sectional study, and the present results should be verified and extended by larger, multicenter longitudinal studies of MSA in the future.

## Conclusion

Our study found that 27.3% of patients with MSA had EDS, and the non-motor dysfunction including fatigue, anxiety, depression, cognitive dysfunction, and sleep-related breathing disorders were associated with an increased risk of EDS. These results may suggest that the difference of involved brain area between patients with and without EDS. Further study on the anatomic pathologic mechanism underlying EDS in MSA is needed.

## Data Availability Statement

The original contributions presented in the study are included in the article/Supplementary Material, further inquiries can be directed to the corresponding author/s.

## Ethics Statement

The studies involving human participants were reviewed and approved by the West China Hospital Clinic Research Ethics Committee. Written informed consent to participate in this study was provided by the patient/participants or patient/participants' legal guardian/next of kin.

## Author Contributions

HW collected data, conducted statistical analysis, interpreted the data, and writing the manuscript. XT conducted statistical analysis and interpreted the data. JZ and YX collected data, conducted statistical analysis, and revised the manuscript. All authors contributed to the article and approved the submitted version.

## Funding

This work was supported by the Project of Sichuan Science and Technology Agency (grant no. 2020YJ0279), Key Research and Development Project of Sichuan Province (grant no. 2020YFS0259), and Miaozi Project in Science and Technology Innovation Program of Sichuan Province (grant no: 2020JDRC0057).

## Conflict of Interest

The authors declare that the research was conducted in the absence of any commercial or financial relationships that could be construed as a potential conflict of interest.

## Publisher's Note

All claims expressed in this article are solely those of the authors and do not necessarily represent those of their affiliated organizations, or those of the publisher, the editors and the reviewers. Any product that may be evaluated in this article, or claim that may be made by its manufacturer, is not guaranteed or endorsed by the publisher.
