# Supplementary material for: Excessive Daytime Sleepiness Is Associated With Non-motor Symptoms of Multiple System Atrophy: A Cross-Sectional Study in China
Source: Front Neurol. 2022 Jan 11;12:798771. doi: 10.3389/fneur.2021.798771 (PMC8786795; doi:10.3389/fneur.2021.798771)
Supplement: Supplementary file 1 [file Table_1.DOCX]

**Supplemental Table 1 Risk factors of EDS in patients with MSA**

|  | **OR (95%CI)** | | |
| --- | --- | --- | --- |
|  | Model 1 | Model 2 | Model 3 |
| FSS | **1.06(1.02-1.10)^*^** | **1.07(1.02-1.10)^*^** | **1.06(1.02-1.11)^*^** |
| MMSE | 0.90(0.81-1.01) | **0.84(0.73-0.98)^*^** | **0.85(0.72-1.00)^*^** |
| HAMA | **1.10(1.02-1.19)^*^** | **1.12(1.03-1.22)^*^** | **1.16(1.04-1.28)^*^** |
| HAMD | **1.10(1.02-1.20)^*^** | **1.12(1.03-1.22)^*^** | **1.13(1.02-1.25)^*^** |
| NMSS | **1.04(1.01-1.06)^*^** | **1.03(1.01-1.06)^*^** | **1.04(1.01-1.07)^*^** |
| Sleep/fatigue | **1.27(1.12-1.43)^*^** | **1.29(1.13-1.48)^*^** | **1.25(1.09-1.45)^*^** |
| Mood/apathy | **1.07(1.00-1.13)^*^** | **1.07(1.01-1.15)^*^** | **1.08(1.01-1.17)^*^** |
| Perceptual problems | 1.14(0.97-1.35) | 1.14(0.97-1.36) | 1.14(0.97-1.36) |
| Attention/memory | **1.23(1.05-1.43)^*^** | **1.24(1.06-1.46)^*^** | **1.29(1.05-1.58)^*^** |
| Gastrointestinal | **1.13(1.00-1.27)^*^** | 1.12(1.00-1.27) | 1.17(0.99-1.38) |
| Miscellaneous | 1.08(.098-1.19) | 1.06(0.96-1.18) | 1.06(0.96-1.18) |
| N3, % | 0.68(0.39-1.16） | 0.73(0.43-1.25) | 0.76(0.49-1.15) |
| AHI, /h | 1.02(1.00-1.05) | 1.02(1.00-1.05) | **1.03(1.00-1.10)^*^** |

**Abbreviations: AHI,** apnea–hypopnea index; **CI,** confidence interval; **FSS,** Fatigue Severity Scale; **HAMD,** Hamilton Depression Scale; **HAMA,** Hamilton Anxiety Scale; **MMSE,** Mini-mental State Examination; **NMSS,** Non-Motor Symptoms Scale; **N3,** N3 sleep stage; **OR,** odds ratio.

**Model 1,** crude model；**Model 2,** adjusted for age, sex；**Model 3,** adjusted for age, sex, disease duration, MSA sub-type, and UMSARS score (I, II and IV). *，P＜0.05
